# *Megaselia scalaris* and *Senotainia tricuspis* Infesting *Apis mellifera*: Detection by Quantitative PCR, Genotyping, and Involvement in the Transmission of Microbial Pathogens

**DOI:** 10.3390/insects15100786

**Published:** 2024-10-09

**Authors:** Franca Rossi, Martina Iannitto, Beqe Hulaj, Paola Manocchio, Francesca Gentile, Ilaria Del Matto, Massimiliano Paoletti, Lucio Marino, Luciano Ricchiuti

**Affiliations:** 1 Teramo, Diagnostic Laboratory, Istituto Zooprofilattico Sperimentale dell’Abruzzo e Molise (IZSAM), 86100 Campobasso, Italy; m.iannitto@izs.it (M.I.); paola_manocchio@outlook.it (P.M.); francy1999@gmail.com (F.G.); i.delmatto@izs.it (I.D.M.); m.paoletti@izs.it (M.P.); l.marino@izs.it (L.M.); l.ricchiuti@izs.it (L.R.); 2 Veterinary Laboratory, Food and Veterinary Agency, Industrial Zone, 10000 Prishtina, Kosovo; bhulaj@yahoo.com

**Keywords:** parasitoid flies, *Apis mellifera*, *Megaselia scalaris*, *Senotainia tricuspis*, qPCR detection, occurrence in apiaries, genotyping, pathogen carriage

## Abstract

**Simple Summary:**

Among the pests infesting the honeybee *Apis mellifera*, the parasitoid flies *Megaselia scalaris* and *Senotainia tricuspis* are not yet well characterized for the occurrence and harmfulness to bee colonies. These flies can attack the forager bee and lay eggs or larvae on its body, which will develop by feeding on the host tissues until it is killed. Their prevalence in different countries is still not well defined, so, in this study, rapid molecular methods were developed to facilitate their detection. The new methods were applied in a preliminary analysis of adult bees and hive debris from parts of Central Italy and the Republic of Kosovo. Both flies were detected in the two countries and more frequently in Italy. The *M. scalaris* flies isolated from Italian apiaries were genotypically related to biotypes from distant countries. Single flies were shown to possibly transmit honeybee microbial pathogens, so their presence should be efficiently contrasted in apiaries.

**Abstract:**

The *Megaselia scalaris* and *Senotainia tricuspis* parasitoid flies of the honeybee *Apis mellifera* were found to infest apiaries of different European and Mediterranean countries but their prevalence and impact on apiary health are little known. Therefore, in this study, quantitative PCR (qPCR)-based methods were developed for their rapid detection directly in hive matrices. The newly developed qPCR assays were targeted at the mitochondrial cytochrome oxidase subunit I (*COI*) gene for the *M. scalaris* and the cytochrome B (*cyt*B) gene for the *S. tricuspis.* The tests were preliminarily applied to 64 samples of adult honeybees and hive debris collected in the Abruzzo and Molise regions, Central Italy, and the Republic of Kosovo showing that both flies occur in the two countries and more frequently in Italy. The positive apiaries in Italy were re-sampled by capturing viable forager bees and isolating emerging flies to carry out the genotyping and analyses aimed at defining if these flies can transmit honeybee pathogens. Genotyping based on the *COI* and *cyt*B gene sequencing for *M. scalaris* and *S. tricuspis*, respectively, identified one *S. tricuspis* genotype and diverse genotypes of *M. scalaris* highly similar to those from distant countries. Some fly isolates harbored the DNA or RNA of honeybee microbial pathogens *Paenibacillus larvae*, deformed wing viruses A and B (DWVA and B), black queen cell virus (BQCV), chronic paralysis virus (CBPV), and *Nosema ceranae*. The results indicated that these parasites should be efficiently controlled in apiaries by using rapid detection methods to facilitate the large screening studies and early detection.

## 1. Introduction

Parasitoid flies represent one of the threats to honeybee (*Apis mellifera*) pollinators worldwide. These flies are known to be present in many countries based on reports of sporadic detection, whereas their prevalence and extent to which they contribute to honeybee colony weakening, are still little known. Diptera able to attack honeybees are the scuttle flies, family Phoridae, *Megaselia scalaris* [1,2,3,4,5,6], and *Senotainia tricuspis*, a viviparous species of the flesh fly family Sarcophagidae, found in Europe, Mediterranean countries and the Middle East [7]. Other Phoridae able to parasitize honeybees were described in particular geographic regions, namely *Apocephalus borealis*, possibly involved in the colony collapse disorder (CDD) in North America [8], and *Melaloncha* spp. in Brazil [9]. Moreover, other *Megaselia* species such as *M. rupifes*, could exert parasitism towards honeybees [10,11,12].

*M. scalaris* is a generalist species able to cause myiases in a variety of living hosts, including humans [13], animals [14], and different arthropod species [15,16,17,18]. Indeed, it was proposed as a biocontrol agent against other pests [14,19]. It also infests inanimate organic matter and food commodities [20]. It was found to infest honeybees in the Abruzzo and Molise regions of Central Italy, with high infestation rates expressed as the ratio of fly larvae number to the number of infested bees [2]. The reports on honeybee infestation by this or other *Megaselia* species regard different European and extra-European countries [3,4,5,6,10,12]. The modeling of *M. scalaris* expansion favored by the ongoing climate changes indicated that this species currently finds optimal living conditions in regions of North and Sub-Saharan Africa, Southern Europe, and France with the potential to invade internal zones of the Balkans and Eastern Europe [21].

*S. tricuspis* was reported to cause heavy infestations with a high colony mortality rate in apiaries. This fly can lead to the collapse of a honeybee colony when it reaches an infestation rate above 70% [22]. *S. tricuspis* flies live in proximity of bee hives where they lie in wait for honeybees leaving for or returning from foraging. Then they attack the bees repeatedly according to a behavior that has been recently described in detail [22,23]. Since each female harbors several hundreds of larvae, it can infest a high number of bees [7]. In Tuscany, Central Italy, adults of *S. tricuspis* emerge during late spring and begin to infest honeybees at the end of May [22]. Larvae formed on bees finally leave the hosts and pupate in the soil. Adults can emerge from the soil either after overwintering, in early spring, or in June-July, since pupae formed in spring develop into adults within 15-20 days giving rise to a second generation in the same year [22].

The extent of the detrimental effects of the parasitoid flies on honeybee health is not yet fully known and requires further investigation. This can also depend on the laborious and time-consuming methods of detection currently used that are based on the observation of fly larvae development on the bodies of honeybees captured and kept alive in controlled conditions of temperature and humidity [2,7]. The time required to complete the analysis is of more than three weeks [2].

Therefore, this study was aimed at developing molecular detection methods based on a quantitative polymerase chain reaction (qPCR) to speed up the detection of parasitoid flies on apparently healthy honeybees. This could allow the detection of the infestation in an early phase and adopt countermeasures such as placing traps to reduce the fly infestation levels [22]. The detection methods developed in this study were characterized for specificity, inclusivity, and limit of detection (LOD) and were preliminarily applied to samples of both adult bees and hive debris collected in the Abruzzo and Molise regions of Central Italy and the Republic of Kosovo.

In addition, thirteen isolates of *M. scalaris* obtained from apiaries in Abruzzo and Molise were genotyped by sequencing a region of the mitochondrial cytochrome oxidase I gene *COI* and compared to biotypes from other world regions to obtain indications on genetic relatedness. This was not possible for *S. tricuspis* because of the unavailability of sequences for comparison.

Finally, fly isolates were analyzed for the presence of honeybee microbial pathogens to investigate their role in the transmission of infectious diseases.

## 2. Materials and Methods

### 2.1. Collection of Bees and Hive Debris

The samples of honeybees and hive debris were collected by beekeepers from 16 apiaries in the Abruzzo and Molise regions of Central Italy and 16 apiaries in the Republic of Kosovo during June–July 2021. The sample sites were uniformly distributed in the two geographical districts. Only one hive per apiary was sampled. At least 30 foraging bees to be examined by molecular tests were captured at the hive entrance and introduced in sterile 50 mL centrifuge tubes (Biosigma, Cona, VE, Italy). The same tubes were used to collect hive debris picked with sterile disposable spoons (Biosigma) from the hive bottom drawer. The samples were transported to the laboratory in refrigerated conditions within 48 h and stored at −80 °C before analysis. The samples collected in the Republic of Kosovo were soaked in RNAlater solution (Thermo Fisher Scientific, Rodano, Italy) before being transported by hand to the Istituto Zooprofilattico Sperimentale dell’Abruzzo e del Molise (IZSAM), branch of Campobasso, Italy, and analyzed.

Viable honeybees were collected in apiaries in Abruzzo and Molise in July 2022 (the sampling of viable bees could not be carried out in the year 2021 because of the COVID-19 pandemic activity limitations) and analyzed for the development of parasitoid flies, as described by Ricchiuti et al. [2].

### 2.2. Design of qPCR Tests Specific for M. scalaris and S. tricuspis

The experimental plan of the present study, as reported in Figure S1, included as the first phase the development of qPCR assays specific for *M. scalaris* and *S. tricuspis*. The PCR primers and probes used in this study were selected after a search of the nucleotide sequences available for *M. scalaris* and *S. tricuspis* in the public domain database accessed through the National Center of Biotechnology Information (https://www.ncbi.nlm.nih.gov/, accessed on 30 August 2024). The representatives of the gene sequences available were aligned by Blastn (https://blast.ncbi.nlm.nih.gov/Blast.cgi?PROGRAM=blastn&PAGE_TYPE=BlastSearch&LINK_LOC=blasthome, accessed on 31 August 2024) to the nucleotide database limiting the search to *M. scalaris* or *S. tricuspis* taxa, respectively, so conserved regions could be identified for the species. Then all the oligonucleotides that could be designed in the conserved regions were aligned by Blastn to verify inclusivity for the highest number of biotypes and exclusivity towards other insect species. The Blastn alignments were carried out for up to 1,000 database entries. The oligonucleotides and probes were provided by Eurofins Genomics (Ebersberg, Germany). Those used in the test specific for *M. scalaris* were targeted on the mitochondrial *COI* gene and were MegaF2: 5′-ACTCTTTTATTAGCAAGAAGTA-3′, MegaR2: 5′-ATAGAAGAAATTCCGGCAA-3′, and MegaP2: 5′Cy5-TTCTAGAATTGCYCATAG-MBGEQ-3′, while those used in the test specific for *S. tricuspis* were targeted on the mitochondrial *cyt*B gene and were StrF1: 5′- GTTACTCCCGTTCACATT-3′, StrR: 5′-TATTGTTCAGAAATAGATTTTATTAATT-3′, and StrP3: 5’FAM-CTTCGTTCAATCCCCAAT-MBGEQ-3’. Other primers designed for sequencing purposes on the same genes were MegaF3: 5’-TAAGTATTATAATTCGAGCTGAA-3’ for *M. scalaris* and senseqF: 5’-AGATAATGCAACACTTACC-3’/senseqR: 5’-GTAAATTATCTCACCATTTAAC-3’ for *S. tricuspis*. For the amplification of the DNA extraction control the primers specific for *Tenebrio molitor* reported by Rossi et al. [24] were used.

### 2.3. DNA and RNA Extraction

The DNA extraction was carried out by two procedures differing in the amount of sample processed and, consequently, amounts of reagents and number of phases. In particular, two or twenty bees and 200 μL or 3 mL of hive debris were examined to ensure an acceptable sensitivity. The hive debris was measured approximately in volume because of the difficulty of manipulation for precise weighing and for the great variability in density among samples. The extraction procedure from two bees or 200 μL of hive debris was designated as “small-scale” and that from 20 bees or 3 mL of hive debris as “large-scale”. In the small-scale procedure, the sample was transferred into a 2 mL Safe-Lock Eppendorf Tube (Eppendorf, Milan, Italy) containing approximately 200 μL of unwashed glass beads of 200 µm diameter (Merck, Darmstadt, Germany) previously sterilized by autoclaving. The bee sample was crushed with a sterile pipette to facilitate the subsequent mechanical disruption by the bead beating. The sample of 200 μL of Macherey-Nagel lysis buffer T1 (Carlo Erba, Cornaredo, Italy) and 25 µg of the DNA extraction control constituted by homogenized *T. molitor* as described by Rossi et al. [24] were combined and the sample was disrupted in TissueLyser II (Qiagen, Milan, Italy) at 30 Hz for 3 min. The sample homogenate was centrifuged at 12,000× *g* for 10 min and the supernatant was transferred into a 2 mL Safe-Lock Eppendorf Tube containing 200 μL of Macherey-Nagel B3 buffer. After mixing, 210 μL of pure molecular biology grade ethanol was added and the DNA was allowed to precipitate for 10 min at room temperature. The whole sample suspension was transferred on a Macherey-Nagel NucleoSpin Tissue kit spin column (Carlo Erba) and DNA purification was completed according to the instructions. The DNA was eluted in two steps in a final volume of 60 μL by adding in each step 30 μL of elution buffer to the column.

In the large-scale procedure, the sample was transferred in a 5 mL screw-cap tube (Sarstedt Srl., Trezzano sul Naviglio, MI, Italy) containing approximately 1 mL of sterile unwashed glass beads of 200 µm diameter (Merck). To the 20 bees, 1 mL of Macherey-Nagel lysis buffer T1 (Carlo Erba) was added, and the bees were crushed with a sterile pipette. An additional 500 μL of lysis buffer T1 and 25 μg of the DNA extraction control were added and the sample was mechanically disrupted in the TissueLyser II instrument equipped with a 5 mL tube adapter. To the hive debris, 1.5 mL of T1 buffer and 25 μg of the DNA extraction control were added prior to disruption. The homogenate was then centrifuged at 12,000× *g* for 10 min and the supernatant was transferred into a 5 mL Eppendorf Tube containing 1.5 mL of B3 buffer. For the hive debris, the centrifugation was repeated by transferring the supernatant in a 5 mL Eppendorf Tube if the supernatant was not clear before mixing with B3 buffer. The sample suspension was mixed and 1.575 mL of pure ethanol was added. The DNA was allowed to precipitate for 10 min at room temperature and the sample was centrifuged at 14,000× *g* for 10 min. Most of the supernatant was discarded by gently aspiring with a micropipette and about 600 μL of it was left at the bottom of the tube. As for the small-scale extraction method, after the pellet resuspension, the sample was loaded on a spin column and the DNA purification was completed.

The small-scale extraction method was applied to six *M. scalaris* flies or one *S. tricuspis* fly to detect honeybee pathogens *Paenibacillus larvae*, *Melissococcus plutonius*, and *Nosema ceranae*.

The RNA was extracted from six *M. scalaris* flies or one *S. tricuspis* fly by the same procedure applied to the honeybees for virus detection by Rossi et al. [24].

### 2.4. Determination of the Limit of Detection (LOD)

The minimum number of target gene copies spiked in samples of bees and hive debris and detectable in 95% of the extraction replicates, i.e., the limit of detection (LOD) [25], was determined by using plasmid pUC57 containing synthetic DNA fragments (GenScript Biotech, Rijswijk, The Netherlands) with sequences identical to the gene regions between the primers. These fragments were quantified by the Qubit 3 Fluorometer (Thermo Fisher Scientific, Rodano, Italy) and the Qubit DNA HS Assay Kit (Thermo Fisher Scientific) according to the manufacturer’s instructions and were used to spike the honeybee or hive debris samples, which, in preliminary assays, tested negative in the qPCR assays to be evaluated in this study.

The LOD was determined also in terms of weight of fly per g of sample by spiking the honeybee and hive debris samples previously found to be negative for the fly-specific tests, with known amounts of *M. scalaris* or *S. tricuspis* homogenate obtained from the isolates made available in this study. The twenty replicates of spiked samples were used for DNA extraction with the two procedures described above.

### 2.5. qPCR Conditions

The quantitative PCR reactions were carried out in a QuantStudio 5 instrument (Thermo Fisher Scientific) with a PCR program comprising an initial denaturation at 95 °C for 5 min and 50 cycles of denaturation at 95 °C for 15 sec, and an annealing/elongation at 52 °C for 1 min. The qPCR reaction contained 1× Takara Premix Ex Taq Probe qPCR (Diatech LabLine, Jesi, AN, Italy), bovine serum albumin (Merck) 0.1 μg/μL, primers and probes for the target 0.2 μM, and primers and probes for the DNA extraction control 0.1 μM and 10 μL of DNA extract in a total reaction volume of 50 μL. The quantitative PCR tests were carried out to detect the microbial pathogens of honeybees in the fly isolates by previously reported tests. These comprised specific tests for *Paenibacillus larvae*, *Melissococcus plutonius*, *Nosema ceranae*, *N. apis,* acute paralysis bee virus (ABPV), black queen cell virus (BQCV), chronic bee paralysis virus (CBPV), deformed wings virus A and B (DWVA and DWVB), and sacbrood virus (SBV) [24,26,27,28]. Synthetic DNA or RNA were used as positive controls in the PCR reactions and the negative amplification controls were represented by extracts obtained without adding a sample.

### 2.6. Sanger Sequencing

Sanger sequencing was carried out by Eurofins Genomics on the amplification products from the *M. scalaris COI* gene obtained with primers MegaF3: 5′-TAAGTATTATAATTCGAGCTGAA-3′ and MegaR2, and from the *cyt*B gene of *S. tricuspis* with the primer senseqF: 5′-AGATAATGCAACACTTACC-3′/senseqR: 5′-GTAAATTATCTCACCATTTAAC-3′. The same oligonucleotides were used as sequencing primers. The obtained sequences received the GenBank accession numbers PQ242635—PQ242647 for *M. scalaris* and PQ255542—PQ255544 for *S. tricuspis*.

### 2.7. Construction of a Phylogenetic Tree

For *M. scalaris* a phylogenetic tree was constructed including the sequences obtained in this study and one representative for each sequence variant retrieved by Blastn. The sequence variants to be included were selected by visually observing each pairwise matching in Blastn. For the tree construction the online tool “advanced phylogeny analysis”, available at http://www.phylogeny.fr/simple_phylogeny.cgi, accessed on 31 August 2024, was used by choosing the functions “Phylogeny analysis” and ”Advanced”, which perform a multiple alignment using MUSCLE, an alignment curation using Gblocks, a construction of the phylogenetic tree by PhyML in default Approximate Likelihood-Ratio Test (aLRT), and a visualization of the phylogenetic tree by TreeDyn.

### 2.8. Statistical Analyses

The distribution of Ct values obtained at LOD for the samples artificially inoculated with *M. scalaris* and *S. tricuspis* was graphically represented by using PAST v4.03 statistical analysis software.

## 3. Results

### 3.1. Performance of the qPCR Methods in the Detection of M. scalaris and S. tricuspis

The GenBank database search for nucleotide sequences from the species *M. scalaris* and *S. tricuspis* retrieved 170 partial or complete sequences of the *COI* gene, one complete mitochondrion genome, and a whole genome in the stage of unassembled and not annotated scaffolds for *M. scalaris*. For *S. tricuspis* only four partial coding sequences for genes elongation factor 1-alpha (Ef1a) and mitochondrial genes *cyt*B, *COI*, and NADH dehydrogenase subunit 4, *ND4*, obtained in a phylogenetic study [29], were available. The Blastn analysis showed that the *M. scalaris* specific qPCR test designed by Furui et al. [20] is targeted at the mitochondrial *ATP6* gene for which only one representative is available in the GenBank database. Since no analysis of inclusivity was possible for this gene, it was decided to design a new test on the *COI* gene, for which many database entries are available.

For *M. scalaris* the first half of the *COI* gene was elected as the region for primer design since it exhibits higher variability among the species of Diptera as well as intraspecies. Moreover, the high number of sequences available for this gene could allow a reliable assessment of the inclusivity of the oligonucleotides. It was not possible to evaluate the inclusivity of the nucleotides designed for *S. tricuspis* because of the unavailability of sequences from different individuals.

None of the oligonucleotides evaluated for *M. scalaris* were specific only for this species, but the combination of MegaF2 with MegaR2 can ensure specificity since these primers differ in the fly species matched beyond *M. scalaris*. Primers targeted at *S. tricuspis* mitochondrial *cyt*B, StrF1 and StrR, appeared to be specific for *S. tricuspis* in in silico evaluation.

The sensitivity of the PCR tests was evaluated with the DNA extracts from the honeybee and hive debris samples contaminated with known copy numbers of plasmid pUC57 containing the synthetic DNA fragments identical to the target regions. In the small-scale extraction procedure, 100 copies of the target per g of sample were detected in all 20 replicates for both target organisms and both sample types. The lowest copy number detected in all replicates of the sample types with the large-scale extraction was 10 copies of the target sequence per g of sample. The cycle threshold (Ct) values at the LOD in the large-scale extraction, which showed higher sensitivity compared with the small-scale extraction method, varied between 30.26 and 32.16 for *M. scalaris* and between 28.36 and 30.02 for *S. tricuspis*.

### 3.2. Analysis of Samples of Honeybees and Hive Debris for the Presence of M. scalaris and S. tricuspis

**Figure 1:**
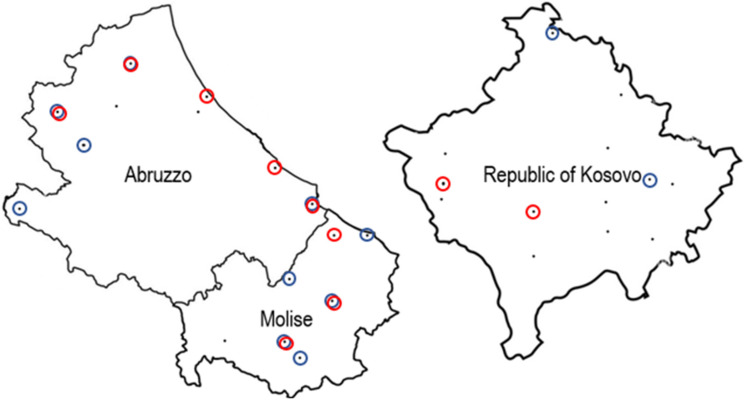
Sites of apiaries sampled in Abruzzo, Molise, and the Republic of Kosovo in June–July 2021. Black dots indicate the sites, blue circles indicate the sites positive for *M. scalaris*, and red circles those positive for *S. tricuspis*.

The samples used for the detection of *M. scalaris* and *S. tricuspis* naturally present in the honeybees and hive debris were obtained from the sampling sites shown as black dots in Figure 1 in Abruzzo, Molise, and the Republic of Kosovo. The sites in which the target organisms were detected are circled.

The samples were analyzed by using the large-scale extraction procedure and the Ct values were in most cases below the range of those observed at the LOD, indicating a low amount of fly material present on the apparently healthy honeybees and hive debris. The Ct values obtained for samples are shown in Table 1, which also reports the towns of collection and sample type.

It can be observed that both flies were more frequently detected in Abruzzo and Molise with 52.25% of the samples positive for *M. scalaris* and 31.25% for *S. tricuspis*. In Abruzzo and Molise the flies were also detected in hive debris in percentages of 37.5% for *M. scalaris* and 12.5% for *S. tricuspis*. In half of the sites positive for *M. scalaris*, the fly was detected both in bees and hive debris, while this was observed only for one among eight sites positive for *S. tricuspis*. For the samples from the Republic of Kosovo the flies were only detected in bees at percentages of 12.5% each. The results indicate that these organisms are more reliably detected using bees as sample type.

### 3.3. Isolation of Flies from Honeybees Captured in Positive Sampling Sites

Samples of viable bees were collected one year later from the molecular analyses in Abruzzo and Molise sites, in which *M. scalaris* and *S. tricuspis* were detected. Emerging fly larvae were observed for five of the samples, namely those taken in Oratino, Termoli, Montenero di Bisaccia, and Trivento for *M. scalaris,* and Vasto for *S. tricuspis*. For each sample four individual flies of those developed in adults were used for the DNA extraction, amplification and sequencing of a *COI* gene region and a *cyt*B region for *M. scalaris* and *S. tricuspis*, respectively. A total of thirteen sequences could be obtained for *M. scalaris* and three for *S. tricuspis*. The results show sequence variability for *M. scalaris* isolates from the same sample in two instances, though most of the isolates shared identical sequences. The three sequences obtained for *S. tricuspis* were identical but divergent from the only database entry available.

**Figure 2:**
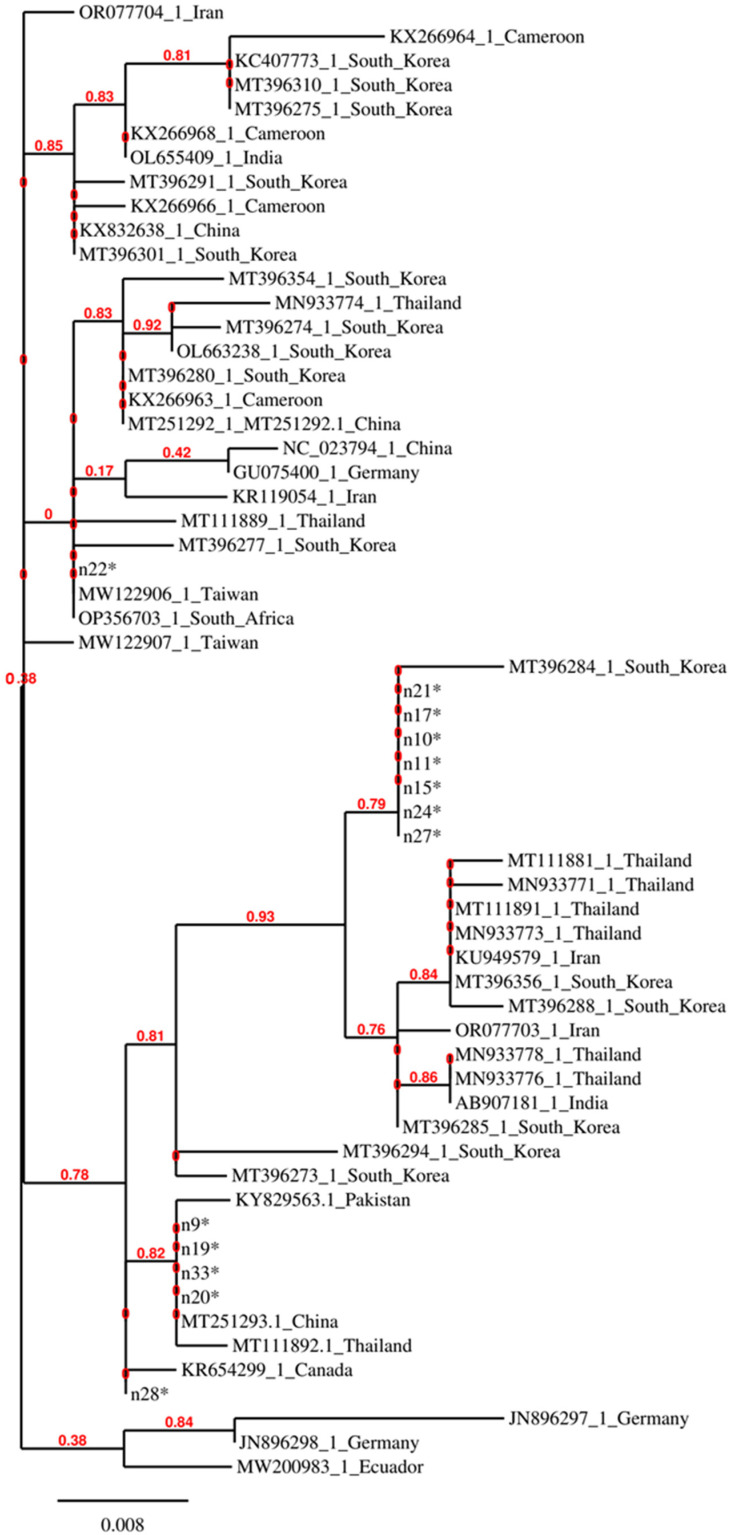
Phylogenetic tree constructed with partial *COI* sequences of *M. scalaris* isolates from this study (labelled with an asterisk) and one representative of each sequence variant retrieved by the GenBank database.

Figure 2 shows a phylogenetic tree constructed with the *COI* gene regions of *M. scalaris*, in which the isolates from Abruzzo and Molise are compared with isolates from other countries.

It can be observed that some isolates from this study, i.e., those that do not have an accession number in Figure 2, do not cluster closely and some of them are identical to biotypes from very distant geographical regions. Identity was also observed among other isolates from distant geographical regions, indicating the possibility that the *M. scalaris* flies might be transferred among countries through infested materials.

### 3.4. Re-Assessment of LOD in Terms of Weight of Fly Material per g of Sample

The availability of fly isolates allowed a better simulation of real sample analysis by using known weights of fly material to spike the bee and hive debris samples negative for the fly-specific qPCR tests. In this case, 10 µg of fly per g of bees or hive debris could be detected in 90% of replicates processed with the small-scale extraction method with Ct values between 30.56 and 33.24 for *M. scalaris* and 27.91 and 30.02 for *S. tricuspis.* With the large-scale extraction method 1 µg per g of bees or hive debris was detected in 95%/100% of the samples processed with Ct values between 31.37 and 34.01 for *M. scalaris* and 29.71 and 30.45 for *S. tricuspis*. Therefore, the large-scale extraction method proved again more sensitive and reliable than the small-scale extraction method for the detection of parasitoid flies in real samples of hive matrices, since in the preliminary analysis most field samples presented Ct values higher than the Ct values at the LOD of the large-scale method (Table 1).

**Figure 3:**
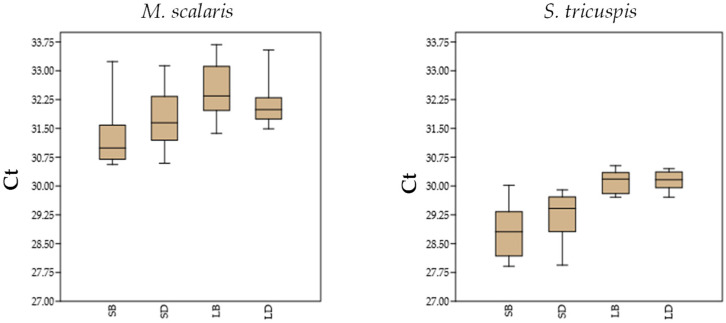
Distribution of the Ct values observed at LOD for *M. scalaris* and *S. tricuspis* isolate materials for the small-scale DNA extraction from honeybees (SB) and hive debris (SD) and the large-scale DNA extraction from honeybees (LB) and hive debris (LD).

Figure 3 shows the distribution of the Ct values at the LOD for 20 samples of honeybees and 20 of hive debris artificially inoculated with known amounts of *M. scalaris* and *S. tricuspis* fly isolates and submitted to DNA extraction with the small-scale and the large-scale extraction methods. The Ct values considered for the small-scale DNA extraction method regard the LOD for 90% positive samples (LOD_90_).

### 3.5. Detection of Honeybee Microbial Pathogens on Parasitoid Flies

The DNA and RNA extracted with the small-scale extraction method from the pools of adult flies that emerged from the bees were used to analyze the presence of honeybee microbial pathogens in the parasites. *P. larvae* was detected in one *M. scalaris* sample, DWVA in four *M. scalaris* samples, BQCV and CBPV in two *M. scalaris* samples, and DWVB in one *M. scalaris* sample. *N. ceranae* was detected in two *S. tricuspis* samples. Viral pathogens and *P. larvae* were present at low levels, close to or below the respective LODs [24,26], and *N. ceranae* was detected with rather high Ct values, namely 34.78 and 35.02, thus at low levels, in the flies. Despite the levels of the detected pathogens being low, their presence indicated that the parasitoid flies could participate in their spread in apiaries.

## 4. Discussion

This study made available experimental procedures suitable to detect the parasitoid flies *M. scalaris* and *S. tricuspis* in hive matrices. For the *M. scalaris* a qPCR test targeted at the mitochondrial *ATP6* gene was described before the one designed in this study [20], but the availability of just one sequence for this gene did not allow the analysis of inclusivity. Moreover, the previously available test was evaluated for specificity towards insects contaminating food commodities and not toward hive-associated insects [20]. For the new test targeted at the *COI* gene, the inclusivity and specificity could be evaluated in silico and appeared to be selective for the *M. scalaris* based on the database entries available at the time this study was carried out. No qPCR tests specific for the *S. tricuspis* were available before this study and, at this time, in silico evaluations excluded possible cross-reactions of the designed primer/probe system with other organisms.

The preliminary application of the developed methods to real samples indicated that the early detection of the infestation on apparently healthy honeybees is possible but the low levels of contamination observed, often below the LOD of 1 µg of fly material per g of sample, indicated that the large-scale DNA extraction method provides more reliable results by reducing the number of false negative results. The low levels of fly material detected on real samples are explainable by the fact that when the infestation is still not manifest only fly eggs or very small fly larvae are present on the bee’s body.

Though the contamination levels were low, the developed methods showed some value in real sample analysis from distinct geographical contexts. A high percentage of sites infested by the flies was identified in the Central Italy regions, Abruzzo and Molise. The lower positive sample percentage identified in the Republic of Kosovo agrees with the distribution model of the infestation of apiaries by the *M. scalaris* proposed by Abou-Shaara and Darwish [21], which predicted a lower current prevalence of this pest in the internal Balkan countries compared to Italy. The presence of the *S. tricuspis* in that geographical region was also detected, so attempts to enlarge monitoring and isolate both flies in that region are worthwhile to understand if it is opportune to adopt measures to contrast their detrimental effects and further spread.

The methods suitable for targeted phylogenetic analyses of the *M. scalaris* and *S. tricuspis* that can be applied to a large number of isolates were devised in this study by designing end-point PCR primers specific for these flies. The tests can be used both for detection and to sequence the intraspecies discriminating regions of genetic markers. Indeed, in previous phylogenetic studies universal primers targeting *COI* or *cyt*B genes were used for the *M. scalaris* and *S. tricuspis* genotyping, respectively [4,17,18,29,30,31,32,33,34,35,36,37]. In particular, the primers specific for the *M. scalaris* reported here could be adopted for targeted studies on the occurrence and genotyping of this fly in fields other than apiculture.

The sampling of fly isolates by the method described by Ricchiuti et al. [2] showed the occurrence of the flies only in four out of the 16 samples sites that were positive in the qPCR-based tests, possibly because a new fly infestation was somehow hindered or because the latter has a higher sensitivity. Despite the low number of flies isolated, it was possible to obtain indications on their genotypic diversity. The possibility to exploit the sequenced gene regions in epidemiological studies was highlighted. Future investigations could be aimed at enlarging the number of fly isolates to reveal possible relationships between the genotype and the frequency and degree of apiary infestations.

The phylogenetic analysis based on the *COI* gene sequence of the *M. scalaris* showed that this generalist species in the Abruzzo and Molise regions is represented by different biotypes and that some of the biotypes are distributed worldwide. This indication could be exploited to investigate the reasons for the high adaptability of the most widespread biotypes by making available viable representatives and studying their behavior and distribution in the environment.

The detection of honeybee microbial pathogens in the parasitoid flies agrees with previous findings regarding the *A. borealis* and *M. scalaris* [3,8], thus confirming the implication of these pests in facilitating the spread of microbial bee diseases.

In conclusion, this study made available methods for the rapid detection of parasitoid flies in honeybees and hive debris that can be exploited to increase the knowledge of their prevalence and trends in geographic distribution. The application of these methods for early detection at the apiary level will allow the adoption of measures such as the positioning of soil types that enhance fly larvae mortality rate around the apiary and chromotropic traps on the roof of the hives [22].

## Figures and Tables

**Table 1:** Sites of sampling in order of collection date with the results of qPCR for parasitoid flies. Results are expressed as Ct because the assays give a presence/absence response.

Sampling Sites	Ct Values			
	Honeybee Samples		Hive Debris Samples	
	*M. scalaris*	*S. tricuspis*	*M. scalaris*	*S. tricuspis*
Italy				
Isola del Gran Sasso	-	-	-	-
L’Aquila	31.15	30.61	-	-
Termoli	37.18	-	-	-
Montereale	35.51	33.88	-	-
Oratino	35.62	37.12	38.29	
Mirabello Sannitico	34.12	-	36.32	-
Trivento	36.42	-	35.24	-
Morrone Del Sannio	34.37	-	-	28.09
Collecorvino	-	-	-	-
Fossacesia	-	23.15	-	31.14
Vasto	41.22	-	-	28.31
Sant’Agapito	-	-	-	-
Montenero Di Bisaccia	37.41	-	-	-
Torricella Sicura	-	34.37	33.25	-
Silvi	-	-	-	-
Pereto	41.26	-	37.06	-
The Republic of Kosovo				
Viti	-	-	-	-
Peje	-	-	-	-
Prishtine	-	-	-	-
Deqan	-	35.63	-	-
Junik	-	-	-	-
Mitrovice	35.15	-	-	-
Deqan	-	-	-	-
Peje	-	-	-	-
Novoberd	33.11	-	-	-
Prizren	-	-	-	-
Podujeve	-	-	-	-
Peje	-	-	-	-
Viti	-	-	-	-
Lipjan	-	-	-	-
Malisheve	-	38.92	-	-
Malisheve	-	-	-	-

## Data Availability

Data obtained in the experiments carried out in this study are available upon request.

## Supplementary Materials

The following supporting information can be downloaded at: https://www.mdpi.com/article/10.3390/insects15100786/s1, Figure S1: Phases of the experimental plan used in this study.
