# Supplementary figures and images for: Megaselia scalaris and Senotainia tricuspis Infesting Apis mellifera: Detection by Quantitative PCR, Genotyping, and Involvement in the Transmission of Microbial Pathogens
Source: Insects. 2024 Oct 9;15(10):786. doi: 10.3390/insects15100786 (PMC11508623; doi:10.3390/insects15100786)

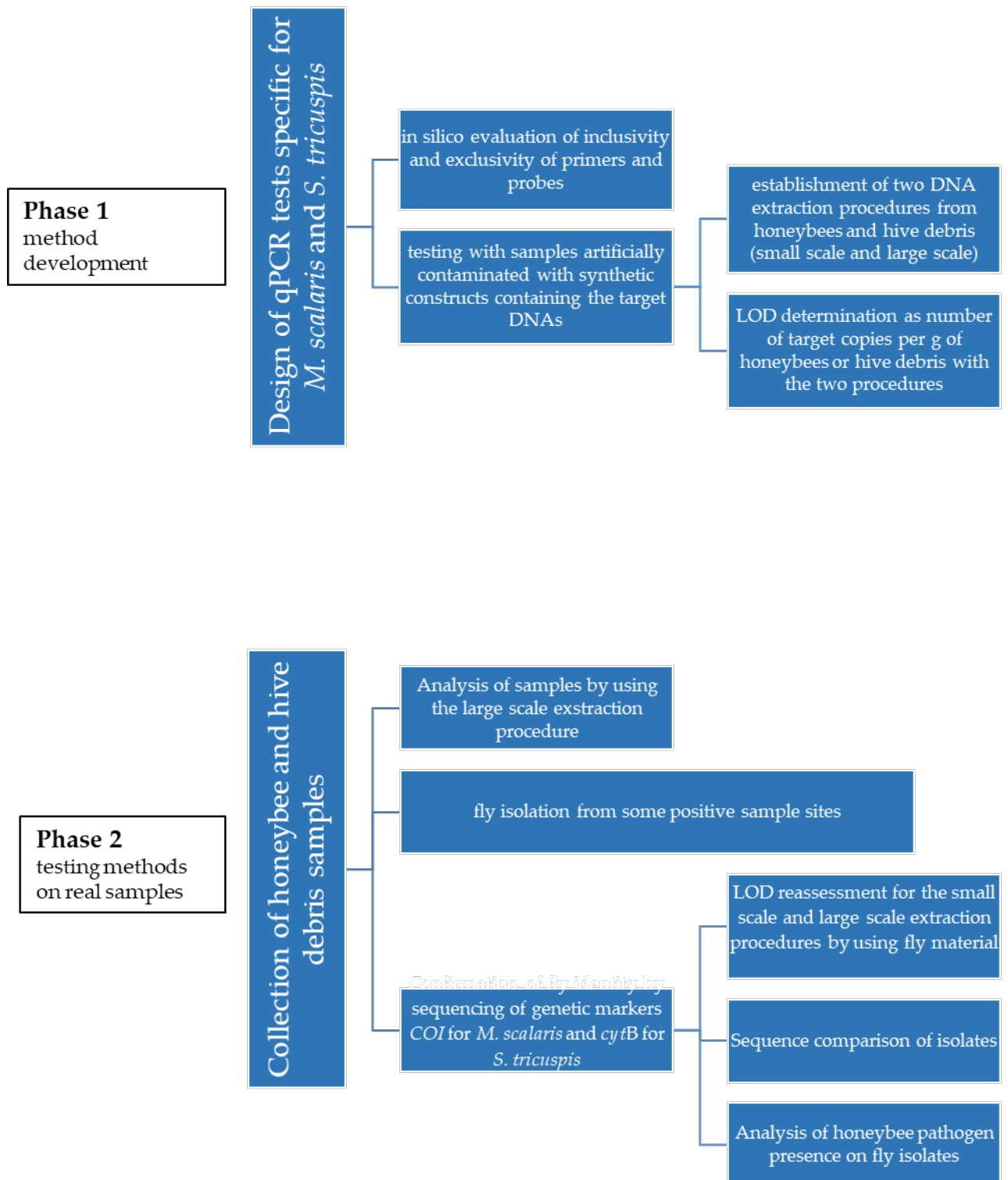

**Figure S1.** Phases of the experimental plan used in this study.

Supplement: Supplementary file 1 [file insects-15-00786-s001.zip › insects-3212792-supplementary.pdf]
